# Inferring heterogeneous transmission and community introduction of antibiotic-resistant bacteria in hospital settings

**DOI:** 10.64898/2026.08.02.26359521

**Published:** 2026-08-04

**Authors:** Jifan Li, Qing Yao, Sen Pei, Ning Ning

## Abstract

Antimicrobial-resistant organisms (AMROs) impose a major burden on healthcare systems, yet routine surveillance cannot readily distinguish colonization imported at admission from transmission acquired within hospitals. This gap is especially consequential because both processes may vary substantially across wards, while asymptomatic carriage, incomplete testing, imperfect diagnostic sensitivity, and patient movement obscure the underlying transmission dynamics. To address this challenge, we developed a blockwise agent-based iterated filter (BAIF) for inference in a patient-level transmission model on a dynamic ward co-location network. The model tracks susceptible and colonized patients as they move across wards, represents unobserved colonization histories, and incorporates the recorded testing schedule and imperfect diagnostic sensitivity. BAIF uses blockwise likelihood evaluation and resampling to estimate ward-block-specific transmission rates and importation probabilities in this high-dimensional latent system. Synthetic experiments showed that BAIF recovered these parameters from partially observed outbreaks. We then applied the framework to hospitalization and microbiological surveillance data collected from 2012 to 2016 at an urban quaternary care hospital in New York City for four AMROs. Transmission and importation were highly heterogeneous across ward blocks. Elevated transmission was repeatedly concentrated in the same ward groups, whereas blocks with the highest importation varied by pathogen. By distinguishing importation-dominated from transmission-dominated ward blocks, the framework can inform more targeted surveillance and infection-control strategies. More broadly, BAIF provides an effective inference framework for high-dimensional, partially observed agent-based models on dynamic contact networks.

**Significance Statement:**

Antimicrobial-resistant organisms can enter hospitals with already-colonized patients or spread after admission, but routine testing often cannot tell these pathways apart. Many carriers have no symptoms, are never tested, or receive imperfect test results, and patients move among wards. We developed a new modeling approach that estimates heterogeneous, ward-group-specific hospital transmission and community introduction from patient movement and surveillance data. Applied to four resistant organisms at an urban quaternary care hospital in New York City, the method revealed large differences among ward groups in both imported colonization and within-hospital spread. Distinguishing these drivers can support more targeted control, including admission screening where importation is high and stronger infection-prevention measures where transmission is elevated.

## Full Text Availability

The license terms selected by the author(s) for this preprint version do not permit archiving in PMC. The full text is available from the preprint server.

